# Beyond hand-crafted features for pretherapeutic molecular status identification of pediatric low-grade gliomas

**DOI:** 10.1038/s41598-024-69870-x

**Published:** 2024-08-17

**Authors:** Kareem Kudus, Matthias W. Wagner, Khashayar Namdar, Julie Bennett, Liana Nobre, Uri Tabori, Cynthia Hawkins, Birgit Betina Ertl-Wagner, Farzad Khalvati

**Affiliations:** 1 Neurosciences & Mental Health Research Program, The Hospital for Sick Children, Toronto, Canada; 2 Institute of Medical Science, University of Toronto, Toronto, Canada; 3 Department of Diagnostic & Interventional Radiology, The Hospital for Sick Children, Toronto, Canada; 4 Department of Diagnostic and Interventional Neuroradiology, University Hospital Augsburg, Augsburg, Germany; 5 Division of Hematology and Oncology, The Hospital for Sick Children, Toronto, Canada; 6 Division of Medical Oncology and Hematology, Princess Margaret Cancer Centre, Toronto, Canada; 7 Department of Pediatrics, University of Toronto, Toronto, Canada; 8 Department of Paediatrics, University of Alberta, Edmonton, Canada; 9 Division of Immunology, Hematology/Oncology and Palliative Care, Stollery Children’s Hospital, Edmonton, Canada; 10 Paediatric Laboratory Medicine, Division of Pathology, The Hospital for Sick Children, Toronto, Canada; 11 Department of Medical Imaging, University of Toronto, Toronto, Canada; 12 Department of Computer Science, University of Toronto, Toronto, Canada; 13 Department of Mechanical and Industrial Engineering, University of Toronto, Toronto, Canada

**Keywords:** Paediatric research, Computer science, Cancer imaging

## Abstract

The use of targeted agents in the treatment of pediatric low-grade gliomas (pLGGs) relies on the determination of molecular status. It has been shown that genetic alterations in pLGG can be identified non-invasively using MRI-based radiomic features or convolutional neural networks (CNNs). We aimed to build and assess a combined radiomics and CNN non-invasive pLGG molecular status identification model. This retrospective study used the tumor regions, manually segmented from T2-FLAIR MR images, of 336 patients treated for pLGG between 1999 and 2018. We designed a CNN and Random Forest radiomics model, along with a model relying on a combination of CNN and radiomic features, to predict the genetic status of pLGG. Additionally, we investigated whether CNNs could predict radiomic feature values from MR images. The combined model (mean AUC: 0.824) outperformed the radiomics model (0.802) and CNN (0.764). The differences in model performance were statistically significant (*p*-values < 0.05). The CNN was able to learn predictive radiomic features such as surface-to-volume ratio (average correlation: 0.864), and difference matrix dependence non-uniformity normalized (0.924) well but was unable to learn others such as run-length matrix variance (− 0.017) and non-uniformity normalized (− 0.042). Our results show that a model relying on both CNN and radiomic-based features performs better than either approach separately in differentiating the genetic status of pLGGs, and that CNNs are unable to express all handcrafted features.

## Introduction

Pediatric low-grade gliomas (pLGG) are a heterogeneous set of tumors with various histological features that can occur anywhere in the central nervous system^1^, and are sometimes referred to as low-grade neuroepithelial tumors. pLGGs account for approximately 40% of central nervous system tumors in children and are the most prevalent brain tumor during childhood^2^. With 10-year overall survival exceeding 90%^3^ death from pLGG is relatively rare. Nonetheless, with a progression-free survival rate of 50%, the morbidity of pLGG is high with many patients needing adjuvant therapy^3,4^.

Recent findings showed that pLGG typically upregulates the mitogen-activated protein kinase pathway, which led to the implementation of targeted therapeutics that can supplement or replace classic cytotoxic treatments^3^. The use of these targeted therapeutics requires accurate detection of the genetic alteration underlying pLGG, which is typically determined through tumor tissue acquired by surgery^5^. In some cases, such as in midline pLGG, it may not be feasible to obtain a biopsy due to the risk of neurologic compromise related to the surgery. Brain biopsies are costly, have numerous risks, leave residual tumor, and occasionally fail due to an insufficient sample.

Radiomics is the process of extracting quantitative features, which can be used to predict characteristics of patients, from radiological images^6^. Over the last decade, radiomics has emerged as a method of decoding tumor phenotypes non-invasively^7–9^. Radiogenomics, a research area within radiomics that combines “radiology” and “genomics”^10^, aims to identify relationships between imaging features and genomic data^11^. Numerous studies have shown the utility of radiomics and radiogenomics in neuro-oncology, including for predicting recurrence, survival, and genetic status^12^.

The two most common genetic alterations in pLGG are KIAA1549-BRAF fusion (BRAF Fusion) and BRAF p.V600E (BRAF Mutation)^13^. Wagner et al. showed that it is possible to differentiate between these two alterations using a machine learning (ML) approach relying on radiomic features extracted from T2-weighted fluid-attenuated inversion recovery (FLAIR) MRI sequences^14,15^. These results demonstrated the feasibility of a non-invasive pLGG genetic alteration identification model. Since then, multiple studies^16–18^ have shown that radiomics can be used to determine the BRAF status of all pLGGs, rather than just differentiating between the two most common genetic alterations. Tak et al.^19^, showed that using Convolutional neural networks (CNNs), an alternative approach to radiomics for extracting information from radiological images, it is possible to accurately sort pLGGs into three groups: BRAF Fusion, BRAF Mutation, and non-BRAF altered. Here we evaluated a more advanced approach, relying on both CNNs and radiomics, on the same classification task.

**Figure 1:**
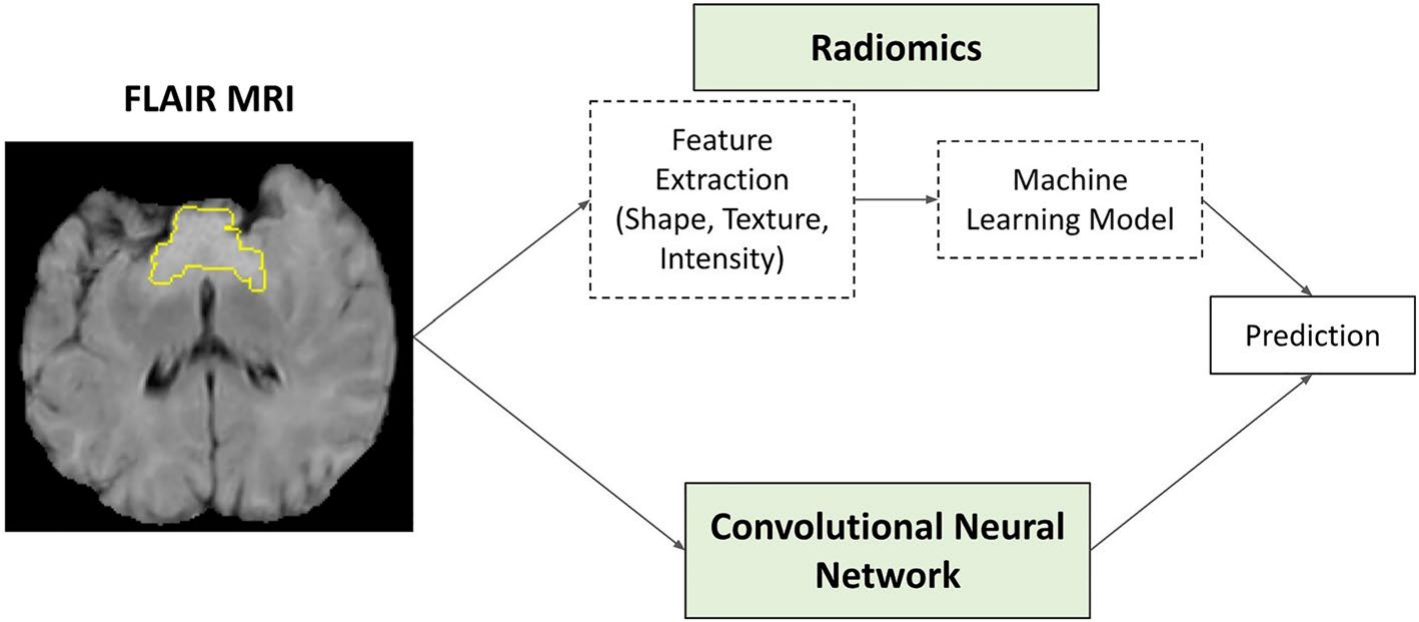
Visualizing the difference between the radiomics and CNN-based approaches for tumor classification. Both take the manually segmented region of interest (ROI) of a pre-processed FLAIR image as inputs. Here the entire brain of a single 2D slice from our dataset is shown, but in practice, a 3D input consisting of just the ROI, which is bounded by the yellow line in the figure, with the rest of the brain zeroed out, was used. The top of the figure shows the radiomics path, which involves extracting features from the image and then using those features to train an ML model to classify the images. The CNN path at the bottom is more direct; the model discovers useful predictive features directly from the ROI.

Radiomics relies on handcrafted features, while CNNs are trained to extract differentiative features (Fig. 1)^20^. The human design of radiomic features limits the amount of useful information that the radiomics approach can extract from medical images^21^. CNNs do not have this limitation, giving them greater expressive power^22^, thus they are often thought to be superior to radiomics^21^. However, CNNs have limitations of their own, namely, they need large datasets to learn from^20,23,24^. Nevertheless, CNNs have exploded in popularity^25^, and have become the dominant method to tackle a variety of medical imaging tasks^26^. The prominence of CNNs for medical imaging tasks in the literature suggests that it is commonly believed that radiomic features are redundant to those discovered by CNNs. According to Orlhac et al., this belief stems from the fact that theoretically, handcrafted features represent only a small subset of the features a neural network can capture^22^. However, in practice, neural networks may have trouble learning to represent certain handcrafted features due to restrictions such as limited data^22^.

Zhang et al. showed that radiomic and CNN features extracted from CT images of pancreatic ductal adenocarcinoma are only weakly related, suggesting a complementary relationship^27^. Other studies have performed more explicit tests and found that CNNs and radiomics work better together for various tasks including the classification of breast tumors^28,29^, ground glass nodules^30^, central nervous system tumors^31^, and adult gliomas^32^. We hypothesized that a combined approach would result in a more accurate pLGG genetic status classification model, thus we aimed to assess the ability of CNNs to complement handcrafted radiomic features. Additionally, we aimed to evaluate whether CNNs can capture all of the information contained in handcrafted features.

## Materials and methods

### Patients and data

All methods of this retrospective study were performed in accordance with the guidelines and regulations of the research ethics board of The Hospital for Sick Children (SickKids) (Toronto, Canada), which approved the study and waived informed consent. The electronic health record database at SickKids was screened for patients treated for pLGG between 1999 and 2018 with pre-therapeutic MR brain imaging and molecular characterization available. All MRIs used in this study were acquired prior to any treatment or intervention. We used FLAIR as our primary imaging sequence because it is useful for assessing both tumor volume and cysts, and was available for the most patients. Furthermore, FLAIR depicts the tumor and surrounding area better than contrast-enhanced T1-weighted images^4^, is known to be sensitive to leptomeningeal spread and non-contrast enhancing lesions^5^, and is considered to be the most sensitive technique to detect brain tumors overall^6^. Thus, of the 397 patients found through initial screening, 45 were omitted due to the absence of a FLAIR image, while 16 others were omitted because the FLAIR image was motion-degraded, leaving 336 patients to be included in this study. Many of these patients also had other sequences available: T2-weighted (265), T1-weighted with (270) and without (260) contrast. All available MRI sequences as well as patient molecular status, age, and sex were manually extracted from the electronic health record database. The demographics of the cohort are summarized in Table 1. Some of these patients (115^14^, 215^15^, 220^33^) were used in previous studies which only accounted for the two most common genetic alterations, resulting in less realistic and clinically useful models compared to this current study.

**Table 1:** Demographics of the patient population.

Total number of patients	336
Patients with BRAF fusion	142 (42%)
Patients with BRAF mutation	70 (21%)
Patients confirmed to not have BRAF Fusion or Mutation (non-BRAF altered)	124 (37%)
NF1	7 (2%)
FGFR 1	13 (4%)
FGFR 2	5 (1%)
Other	45 (14%)
Unidentified	54 (16%)
Histopathology	
Pilocytic astrocytoma	142 (42%)
Ganglioglioma	66 (20%)
Low-grade astrocytoma	40 (12%)
Dysembryblastic neuroepithelial tumor	25 (7%)
Diffuse astrocytoma	21 (6%)
Other	42 (13%)
Median patient age (Interquartile range)	8.6 (4.8, 12.8)
Female	154
Male	182

### Molecular subtyping

A stepwise approach was used for the molecular characterization of pLGG, as previously described in^14^. First immunohistochemistry was used to detect BRAF mutation, then either a nCounter Metabolic Pathways Panel (NanoString Technologies) or fluorescence in situ hybridization was used to identify BRAF Fusion. Samples that were negative for BRAF Mutation and Fusion were analyzed further using other sequencing strategies detailed in^13^, such as RNA sequencing or panel DNA sequencing. For most patients, formalin-fixed paraffin-embedded tissue obtained during biopsy or resection was used for the molecular analysis, otherwise, frozen tissue was used.

### MRI acquisition, segmentation, and preprocessing

Patients underwent brain MRI at 1.5 T or 3 T using either the Achieva, (Philips Healthcare), or Magnetom Skyra (Siemens Healthineers) system. MRI data were deidentified after being extracted from the PACS at SickKids. Each patient had a 2D FLAIR sequence, either axial or coronal.

As described in^14^, tumor regions were identified through segmentation of the FLAIR images by a pediatric neuroradiologist (MWW). Segmentations were validated by a senior neuroradiologist with more than 20 years of experience (BEW). The level tracing-effect tool of 3D Slicer^34,35^ (Version 4.10.2) was used to perform semi automated tumor segmentation. The tumor region alone was used as input to our machine-learning models. Images were z-score normalized, bias-corrected, and isotropically resampled to a resolution of 240 × 240 × 155 using 3D Slicer, to help account for differences in slice thickness, field strength, and pixel spacing.

### Radiomic feature extraction

The same pipeline as in^14^ was used to generate radiomic features from the MRIs. The SlicerRadiomics extension of 3D Slicer was used to access PyRadiomics, an open-sourced package for radiomic feature extraction^36^. Default PyRadiomics settings were used, for example, bin width was set to 25. For each patient, 851 radiomic features, including first-order, shape, and second-order (texture) features, were extracted. The full list of radiomic features can be accessed in the Online Supplemental Data of^14^.

### ML models

**Figure 2:**
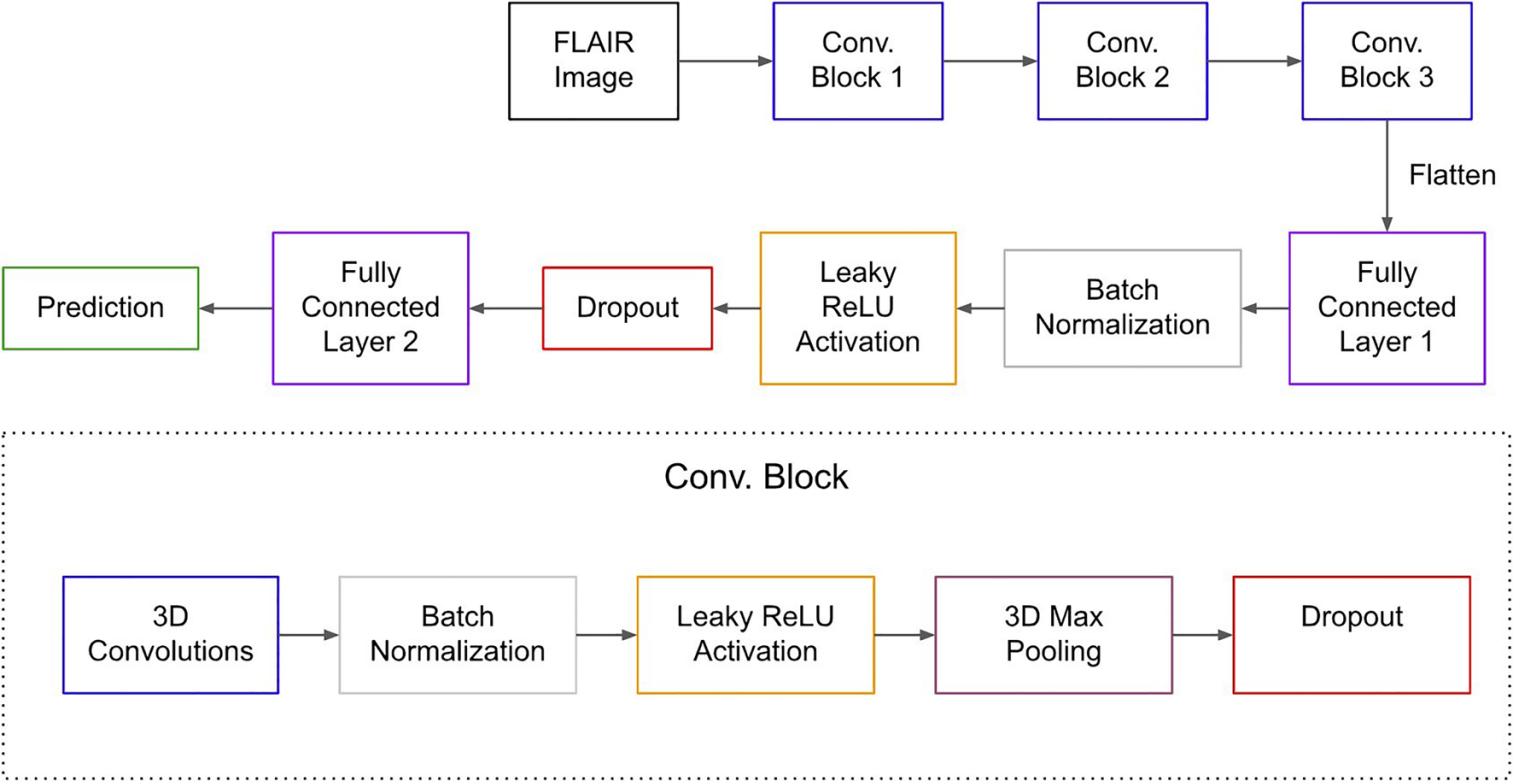
The top of this figure describes the full architecture of our custom shallow 3D CNN. The bottom of the figure describes the contents of the three convolutional blocks (Conv. Block). Convolutional blocks 1, 2, and 3, had 16, 32, and 64 output channels respectively. The first fully connected layer had 64 input and 16 output neurons, while the second fully connected layer had 16 input and 3 output neurons, representing the three classes, BRAF Fusion, BRAF Mutation, and non-BRAF altered.

**Figure 3:**
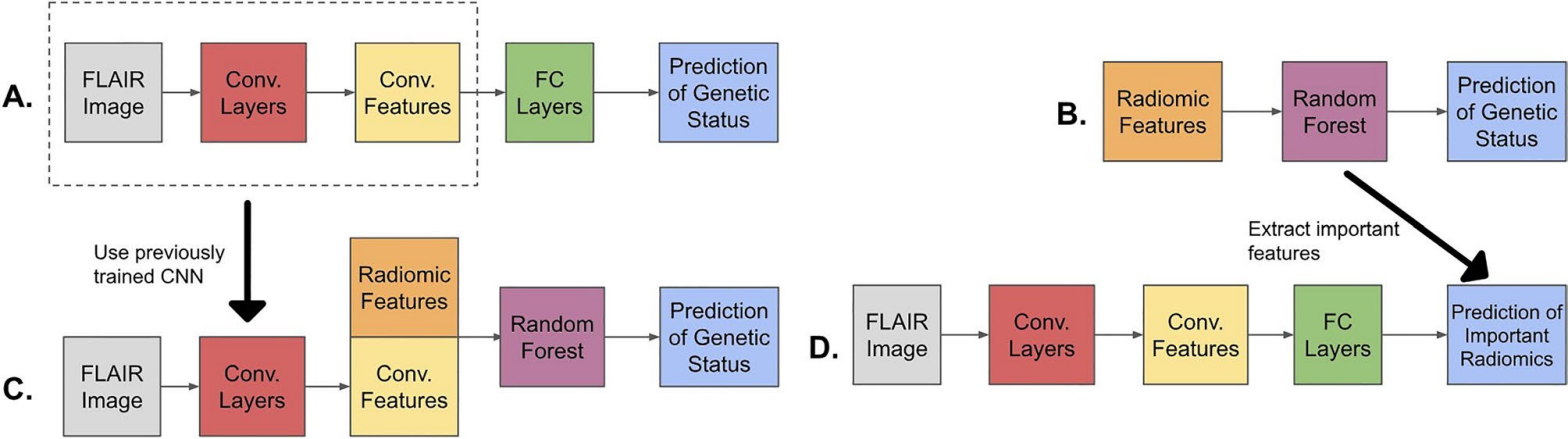
A visualization of our experiments. A (top left) and B (top right) depict the processes of training the CNN and radiomic models. C (bottom left) describes the combined model which takes the convolutional layers from A and uses them to generate CNN features that are then combined with the radiomic features and fed into a random forest model. D (bottom right) illustrates our attempt to learn radiomic features with CNNs from the MRIs. We first sorted the radiomic features according to their permutation importance in the random forest model from B. These features were then used as labels which we then attempted to learn using the same CNN configuration found as in A.

Each of our models was trained on a multiclass classification task with three class labels: BRAF Fusion, BRAF Mutation, and non-BRAF altered, a heterogeneous class containing numerous genetic alterations. The radiomics approach relied on a Random Forest (RF) model. We experimented with two different CNN architectures, a well-established deep neural network, the 3D ResNet^37^, and a custom shallow 3D CNN (Fig. 2) with three convolutional layers and two hidden fully connected layers. For the shallow CNN, the Leaky ReLU activation function and batch normalization were used between each layer, while max-pooling was employed after each convolutional layer. For the combined method we implemented feature-level fusion^23^ similar to the techniques implemented in^27^ and^32^, where CNN features are extracted from the fully-connected hidden layers and then used together with the radiomic features to train an RF (Fig. 3). We trained CNNs on both the FLAIR sequence alone and in conjunction with the other sequences (T2-weighted, T1-weighted with and without contrast), in an attempt to improve the accuracy of the model by including additional information. The only difference between these two configurations was the number of input channels (one versus four), and when training the model on four sequences we employed an input-level dropout approach^38^ to deal with missing sequences since not all patients had every sequence available.

In addition to testing whether CNNs and radiomics were complementary by combining them, we tested whether the CNNs could learn radiomics features as labels of MR images (Fig. 3). It was not computationally feasible to test the ability of the CNN to learn all 851 radiomic features, so we focused on the features that were the most predictive of pLGG molecular status in our radiomics model according to permutation importance. The rationale for using this subset of radiomic features was that if we found that the CNN could not learn them, we would gain some insight into the limitations of CNNs on this classification task. To the best of our knowledge, our experiment was the first to use real MRI images to test the ability of CNNs to learn radiomic features.

Along with the preprocessing steps outlined above that were performed to prepare the MRIs for radiomic feature extraction, each image was registered to the SRI24 atlas^39^ prior to being used with the CNN. Registration is commonly included in CNN pipelines, for instance, the Brain Tumor Segmentation challenge^40–42^ registers images to the SRI24 atlas. Image registration is thought to help CNNs identify positional information by aligning images such that each voxel represents the same anatomical location across all images. The radiomic approach does not account for positional information, which is not captured by typical radiomic features. Thus we did not include registration in the radiomics pipeline, since registration warps and resizes the brain, adding noise to the radiomic features, without any clear benefit.

### Experiment configuration

To build the RF radiomics model we split the patients into test (20%) and development (80%) sets, where the development set is the training set plus the validation set, 25 times and performed five-fold cross-validation within each development set to tune hyperparameters (Table 2). Under this nested-cross validation approach, results were unlikely to be influenced by random data splitting. Due to the high computational load of training neural networks, a nested cross-validation scheme was not feasible for experiments using CNNs. We still repeated our experiments to avoid results biased by (un)lucky data splits by resampling test (20%) and development (80%) sets 25 times for the classification tasks and 10 times when attempting to learn radiomics with CNN. However, we only optimized CNN hyperparameters (Table 2) over a single split of the development set (still into five parts, four of which were used for training, while the last one was used for validation), rather than using five-fold cross-validation. Stratified sampling was used for all data splits, to account for class imbalances. When training CNNs the batch size was set to 16 images, and we used a cosine annealing period^43^ of 50 epochs, with a single warm restart for a total of 100 epochs of training. Kaiming Normal weight initialization^44^ was implemented to initialize weight parameters in both the convolutional and fully connected layers. Dropout was used during model training and switched off for inference. The final CNN used for evaluation on the test set was taken from the best epoch of the optimal set of hyperparameters, as measured by validation loss on the validation portion of the development set.

**Table 2:** Hyperparameters for radiomics RF and CNN models.

Random Forest hyperparameter	Values explored
Number of trees	[75, 150, 300]
Minimum samples per leaf	[1, 2, 4]
Minimum samples for a split	[2, 4, 8]
Maximum depth	[4, 8, 12]
Proportion of samples to use in each tree	[0.5, 0.75, 1.0]
Proportion of features to use in each tree	[0.25, 0.5, 0.75]

CNN hyperparameter	Values explored
Learning rate	[0.1, 0.01, 0.001, 0.0001]
Dropout (fully connected layers)	[0.3, 0.6]
Dropout (convolutional layers)	[0.3, 0.6]

We performed an exhaustive grid search across these hyperparameters to find the optimal model for both our radiomics, CNN, and combined models. For each development/test set split, the optimal model was found through hyperparameter optimization on the development set, and then evaluated on the test set.

Both CNN and RF classification models were trained using cross-entropy loss and evaluated by One-Vs-Rest Area Under the Receiver Operating Characteristic Curve (AUC) on held-out test data. Correlation between predicted and true radiomic feature values was used to evaluate CNNs that were trained, using mean squared error loss, to learn radiomics features as MR image labels. The radiomics, CNN, and combined models were trained and tested on identical data splits. Resampling results in the test set of one trial containing samples from the training set of another trial, which invalidates the independence assumption of the traditional t-test^45^. To account for this dependence, we use the “corrected resampled t-test”^46,47^ to test for statistically significant differences in model performance. Python 3.11.0 was used to run all experiments. We relied upon Python’s Scikit-learn package 1.2.0^48^ to execute ML concepts, while the PyTorch 1.13.0 library^49^ was used to implement deep-learning models.

## Results

The performance of each of the models we trained is summarized in Table 3. On average, using the FLAIR sequence alone, the difference between the performance of the custom shallow CNN (mean AUC: 0.764) and the ResNet (0.758) was not statistically significant (*p*-value: 0.3836). Thus, we proceeded with the more computationally efficient custom shallow CNN, which is 10 × faster than the ResNet. The difference between the performance of the CNN when trained on FLAIR alone and with all four sequences (mean AUC: 0.757) was not significant (*p*-value: 0.272), so we continued with FLAIR alone for the remaining experiments.

**Table 3:** Listed are the mean AUC and 95% confidence intervals for the mean AUC over 25 different resampled test sets for the custom shallow CNN (FLAIR only, and all sequences), ResNet, radiomics, and combined models.

		95% confidence interval of mean	
Model	Mean AUC	Lower bound	Upper bound
Shallow CNN (All)	0.757	0.746	0.769
ResNet (FLAIR)	0.758	0.743	0.772
Shallow CNN (FLAIR)	0.764	0.751	0.777
Radiomics (FLAIR)	0.802	0.785	0.819
Combined (FLAIR)	**0.824**	**0.810**	**0.838**

Best performing model is in bold.

The performance of the radiomics model (mean AUC: 0.802) eclipsed that of the CNN (0.764), but the combined model performed best (0.824). The differences were significant; *p*-values when testing whether the combined model was better than the radiomics model or the CNN alone were 0.0344 and 0.0002 respectively, while the *p*-value when comparing the radiomics model and the CNN was 0.0350.

**Figure 4:**
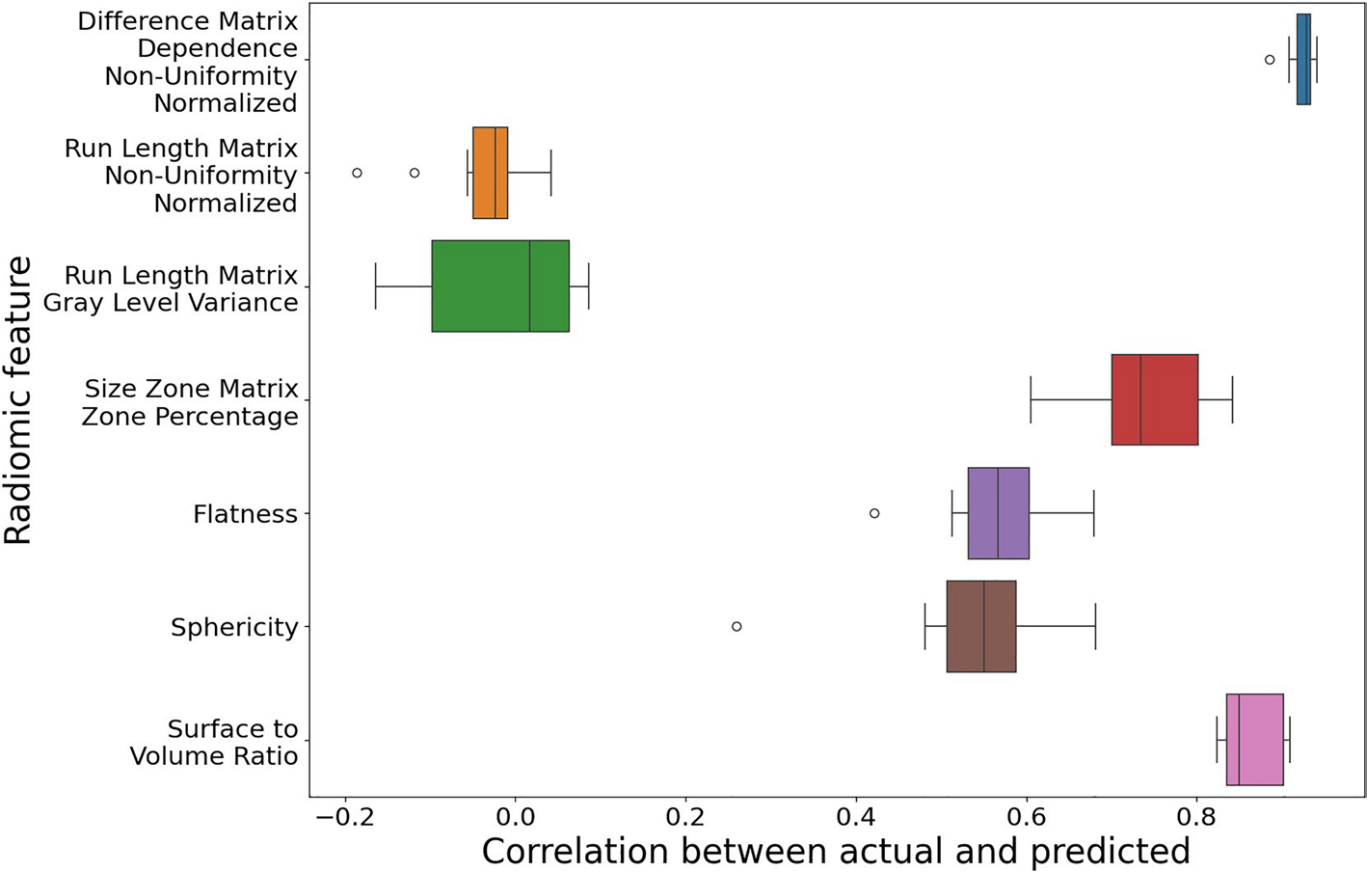
Each boxplot represents the average correlation between actual and predicted radiomic feature values over 10 different splits of the data into development and test sets.

The most important features in the radiomics model according to permutation importance are listed in Table 4. Figure 4 depicts the ability of the CNN to learn these features as labels from the FLAIR MR images. The CNN was able to learn gray level dependence matrix (GLDM) dependence non-uniformity normalized (average correlation: 0.924) and surface-to-volume ratio (0.864) well. It had more trouble, but some level of success, with gray level size zone matrix (GLSZM) zone percentage (0.735), flatness (0.563), and sphericity (0.532). The CNN had no ability to learn gray level run length matrix (GLRLM) non-uniformity normalized (− 0.042) or variance (− 0.017).

**Table 4:** The seven most predictive radiomic features ranked in order of the ability of the CNN to accurately predict the feature value.

Feature name	Category	Description	Average correlation
GLDM dependence non-uniformity normalized	Texture	Similarity of dependence	0.924
Surface-to-volume ratio	Shape	Non-dimensionless measure of sphericity dependent on volume	0.864
GLSZM zone percentage	Texture	Coarseness of the texture	0.735
Flatness	Shape	Ratio of the lengths of the two largest principal components	0.563
Sphericity	Shape	Roundness of the tumor region	0.532
GLRLM variance	Texture	Variance of the gray-level intensity of the runs	− 0.017
GLRLM gray-level non-uniformity normalized	Texture	Similarity of gray-level intensity features	− 0.042

The last column contains the average correlation between the actual feature value and the CNNs prediction.

## Discussion

This study explored the use of radiomics and CNNs to create a model that can non-invasively identify the underlying genetic alteration of pLGGs, labeling them as either BRAF Fusion, BRAF Mutation, or non-BRAF altered. We found that radiomics (mean AUC: 0.802) outperformed CNNs (0.764) and that a combined model performed better than either approach on its own (0.824). Our experiments also showed that utilizing a deeper model did not improve CNN performance. Furthermore, performance was similar with and without additional MRI sequences beyond FLAIR. We trained CNNs to learn predictive radiomic features from MR images and found that the CNN could estimate the value of certain radiomic features well, however, it was completely incapable of predicting others. These results suggest that the CNN may have performed worse than radiomics because it could not extract all the information contained in the radiomic features.

Handcrafted features have fallen out of favor for medical imaging tasks^50^, while neural network-based approaches have grown in prominence, in part because, theoretically, they can learn to discover any handcrafted feature from an image; but this does not always play out in practice. We think that it is too early to give up on the traditional ML techniques relying on handcrafted features. It is yet unclear as to whether the drawbacks of radiomics are more problematic than those of CNNs. There have been only a few studies that compare the two approaches directly, and the results have been inconsistent. CNNs have been shown to outperform radiomics for the identification of schizophrenia^51^, axillary lymph node metastasis^21^, and malignant breast lesions^20^. Radiomics surpassed CNNs for the classification of central nervous system tumors^31^, and the differentiation of malignant and benign ground glass nodules^30^. Our study adds another data point to this limited body of evidence and suggests that, contrary to popular belief, CNNs alone might not always be the best option for medical imaging tasks.

Klyuzhin et al.^24^ found that CNNs can learn first-order intensity and size-related radiomic features but are less able to learn shape irregularity and heterogeneity properties on a synthetic PET dataset. Their findings provided preliminary evidence that CNNs used alone in medical imaging are fundamentally limited due to their inability to capture radiomic features associated with clinical outcomes^22^. Our investigation, performed on MR images from real patients, provides further insights into the relationship between CNNs and radiomic features. In line with the results of^24^, we found that CNNs are better at learning features influenced by size, like surface-to-volume ratio, than shape-based features, like sphericity and flatness. Furthermore, we explored the ability of the CNN to learn texture features derived from the GLDM, GLRLM, and GLSZM. Klyuzhin et al. concluded that CNNs have a limited ability to learn radiomic texture features generally, whereas our results suggest that CNNs have trouble learning only certain pLGG radiomic texture features. GLDM dependence non-uniformity normalized was learned well, though the CNN performed worse on GLSZM zone percentage and had no ability to learn GLRLM variance or gray-level non-uniformity normalized. Overall, our results provide supporting evidence for the conclusion of Klyuzhin et al.^24^, that CNNs are not capable of capturing all of the tumor information contained in handcrafted radiomic features.

There are limitations to this study. First, though our dataset is large from a pediatric neuro-oncology perspective (336 patients), from an ML standpoint, it is relatively small and was collected from a single institution. Prospective data and data from other institutions are needed to evaluate the generalizability of our approach and the reliability of our model. Nevertheless, our dataset is diverse, having been collected over two decades using different MRI scanners and field strengths, and our results were found to be statistically significant under a rigorous nested cross-validation approach. Second, our claims about the performance of CNNs, radiomics, and combined models, the (in)ability of CNNs to learn certain radiomic features, and the lack of benefit when including different MRI sequences beyond FLAIR and larger CNNs, are limited to the specific scenarios we explored in this study. There are countless other combinations of radiomics models, neural networks, experiment configurations and preprocessing techniques, that could result in different conclusions; these need to be explored to evaluate the generalizability of our results. However, our results align with related studies^24,27–32^ that used different configurations, models, and data sources, giving us confidence that our conclusions will be confirmed by future studies. Third, our analysis was based on the entire tumor region. Performance may have been better with more specific labels (edema, necrosis, and enhancing vs non-enhancing structures); this exploration has been left to future work. Finally, it is not clear from our experiments why the CNN underperformed radiomics on the classification task and was unable to learn some radiomic features. Further experiments on larger datasets are required to determine whether CNN performance was limited because of a lack of data, or because there are inherent limitations to the performance of CNNs due to their design.

## Conclusion

In this study, we created a model capable of non-invasively classifying pLGGs by BRAF status. We investigated the performance of radiomics and CNNs both separately and combined for this classification task, and found that the combined model relying on both CNNs and radiomic features performed best. Furthermore, we identified radiomic features that CNNs had trouble learning, uncovering limitations of CNNs in terms of the types of information they can extract from medical images. Future studies with large external and prospective datasets are necessary to improve diagnostic accuracy and further validate the robustness of the results.

## Data Availability

The datasets generated and/or analyzed during the current study are available from the corresponding author on reasonable request pending the approval of the institution(s) and trial/study investigators who contributed to the dataset.
